# A neutral polysaccharide from *Ophiocordyceps lanpingensis* restrains cisplatin‐induced nephrotoxicity

**DOI:** 10.1002/fsn3.2317

**Published:** 2021-05-11

**Authors:** Shubo Zhou, Yongchun Zhou, Jiaji Yu, Li Jiang, Yingying Xiang, Juan Wang, Yaxi Du, Xiuming Cui, Feng Ge

**Affiliations:** ^1^ Yunnan Provincial Key Laboratory of Panax notoginseng Faculty of Life Science and Technology Kunming University of Science and Technology Kunming China; ^2^ Yunnan Cancer Center Molecular Diagnostics Center Yunnan Cancer Hospital & the Third Affiliated Hospital of Kunming Medical University Kunming China; ^3^ Department of Microbiology, Immunology & Molecular Genetics University of California Los Angeles CA USA; ^4^ Department of Stomatology Yan’an Hospital Affiliated to Kunming Medical University Kunming China

**Keywords:** cisplatin, nephrotoxicity, *Ophiocordyceps*, polysaccharide

## Abstract

*Ophiocordyceps lanpingensis* is an edible mushroom distributed over the south‐eastern part of the Tibet Plateau, which is also recognized as an effective ethnomedicine to alleviate diseases. This study explored the effects of a kind of *Ophiocordyceps lanpingensis* neutral polysaccharide (ONP) on RAW264.7 macrophages and cisplatin‐induced nephrotoxicity. The results showed that ONP relieved the inflammatory response of RAW264.7 macrophages by increasing the expression level of anti‐inflammatory factor IL‐10. Furthermore, ONP treatment significantly prolonged the survival of the mice treated by cisplatin through decelerating pathological progress and alleviating damaged functions of the kidneys. Compared with the cisplatin group, ONP reduced the oxidative stress of the renal cells and the expression levels of pro‐inflammatory factors. Apoptosis of renal cells was also weakened in the ONP treatment group. These findings indicated that ONP alleviated cisplatin nephrotoxicity mainly by inhibiting oxidative stress, inflammation, and apoptosis in the kidneys, underscoring the potential of ONP supplementation to alleviate the side effects of cisplatin chemotherapy.

## Graphical abstract

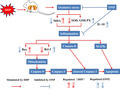

## 1 INTRODUCTION

*Ophiocordyceps lanpingensis* is one strain of edible fungi distributed over the south‐eastern part of the Tibet Plateau (Chen et al., 2013). Minorities living in Tibet Plateau use *O. lanpingensis* as an ethnomedicine for alleviating urinary diseases and health care; and so far, no significant side effect is found in these ethnic minority groups during the usage of *O. lanpingensis*. Nevertheless, the pharmacological mechanisms of *O. lanpingensis* in treating diseases are still unknown.

In recent years, there have been strong interests in the development of new and effective medicines or dietary supplements for the treatment of renal diseases using natural resources, of which mushroom was particularly prominent (Li et al., 2020; Ma et al., 2020; Zhang et al., 2019). Some studies have shown that edible and medicinal mushroom could attenuate kidney diseases, but the effective ingredients and pharmacological mechanism were still unclear (Ma et al., 2017; Zhang et al., 2019). Polysaccharides are considered to be one of the macronutrients, and plant polysaccharides have the potential to treat diseases (Li et al., 2020; Ma et al., 2020). In our previous study, we also found that polysaccharides might be the main bioactive ingredients in *O. lanpingensis* (Zhou et al., 2020).

Cisplatin (cis‐diamminedichloroplatinum II, DDP), which is used to treat more than 20% of cancer patients, is one of the most effective chemotherapy drugs for solid tumors (Dasari and Tchounwou, 2014). However, the usage of DDP‐based chemotherapy is limited by its tissue toxicities, especially the nephrotoxicity (Khairnar et al., 2020). Serious side effects of acute kidney injury (AKI) may be incurred when DDP is administered (Holditch et al., 2019). Giving amifostine before DDP injection is currently considered as an efficient approach to prevent DDP‐induced nephrotoxicity; nevertheless, amifostine also has certain toxicities such as hypotension and nausea/vomiting (Bukowski, 1996). Therefore, it is necessary to explore dietary supplements suitable for alleviating nephrotoxicity induced by DDP with no apparent side effects for cancer patients. Based on the DDP‐induced nephrotoxicity and the traditional usage of *O. lanpingensis* in protecting urinary system, it is intriguing to investigate whether *O. lanpingensis* has pharmacological activity to attenuate DDP‐induced nephrotoxicity.

In this study, we isolated and characterized the *O. lanpingensis* neutral polysaccharide (ONP). RAW264.7 macrophages were used to preliminarily explore the anti‐inflammatory activity of ONP. Furthermore, the effects of ONP on DDP‐induced nephrotoxicity in mice were investigated. The alleviation of DDP‐induced nephrotoxicity by ONP was confirmed based on the changes of physiological and biochemical indices, as well as the histopathological parameters. The mechanisms for the remission of DDP‐induced nephrotoxicity by ONP treatment in mice were discussed, including antioxidative, anti‐inflammation, and anti‐apoptosis activities.

## 2 MATERIALS AND METHODS

### 2.1 Materials

DEAE‐Sepharose^TM^ Fast Flow column was purchased from General Electric Company, Inc (Connecticut, USA). DDP was purchased from Qilu Pharmaceutical Co. Ltd (Shandong, China). The assay kits of blood urea nitrogen (BUN), serum creatinine (CRE), superoxide dismutase (SOD), malondialdehyde (MDA), and glutathione peroxidase (GSH‐PX) were purchased from Nanjing Jiancheng Bioengineering Institute Co. Ltd (Nanjing, China). The micro blood calcium/phosphorus (Ca/P) assay kit was purchased from Solarbio Life Science Co. Ltd (Beijing, China). The primary rabbit and mouse antibodies including NADPH oxidase 4 (NOX4), interleukin‐1 beta (IL‐1β), tumor necrosis factor‐alpha (TNF‐α), caspase‐3, cleaved caspase‐3, caspase‐8, caspase‐9, Bax, Bcl‐2, β‐actin, mitogen‐activated protein kinase 8 (MAPK8, JNK), p‐JNK, mitogen‐activated protein kinase 14 (MAPK14, p38), p‐p38, extracellular regulated protein kinases (ERK1/2), p‐ERK1/2, secondary goat anti‐rabbit, and goat anti‐mouse were purchased from Proteintech Group, Inc (Chicago, USA) and Thermo Fisher Scientific, Inc (Massachusetts, USA). TUNEL kit was purchased from Sigma‐Aldrich, Inc (San Francisco, USA).

### 2.2 Analysis of polysaccharides

The ONP was extracted from *O. lanpingensis* according to the description in previous report (Chen et al., 2016; Zhang et al., 2017; Zhou et al., 2020). The crude polysaccharide was separated and purified according to the previous method, and ONP was obtained after ethanol precipitation and DEAE‐Sepharose^TM^ Fast Flow column purification. The ONP was determined to be a neutral polysaccharide using a pH meter.

The phenol sulfuric acid method was used to determine the total carbohydrate content (Albalasmeh et al., 2013). Fourier transform infrared spectrometer (FT‐IR, Bruker, Germany) was employed to study the main functional groups of the ONP (Wang et al., 2020). FT‐IR measurement was performed on freeze‐dried ONP, and the FT‐IR spectrum was recorded in the range of 4000–400 cm^‐1^.

The composition of monosaccharide was analyzed by ultra‐performance liquid chromatography mass spectrometry (UPLC‐MS) using the 1‐phenyl‐3‐methyl‐5‐pyrazolone (PMP) derivatization method as previously described (Xia, Deji et al., 2020). In brief, the ONP sample was hydrolyzed with 2 M trifluoroacetic acid (TFA) at 110°C for 4 hr, followed by evaporation under vacuum to dryness. The remaining solid was redissolved in water, followed by mixing with equal volumes of 0.5 M PMP methanol solution and 0.3 M NaOH solution, and kept at 70°C for 2 hr. The reaction was stopped by adding 0.3 M HCl and then washed three times with chloroform, and the aqueous layer was collected for UPLC‐MS analysis, which was performed using an Agilent Eclipse C18 column (2.1 × 50 mm, 2.7 μm). The Waters UPLC instrument was used at 25°C. The detection was performed with ammonium acetate buffer (0.02 M, pH 7.0, mobile phase A) and acetonitrile (mobile phase B) at a flow rate of 0.4 ml/min.

The analytes were detected using multireaction monition (MRM) in a positive ionization mode (Waters Xevo TQ‐S Micro, Massachusetts, USA). Electrospray ionization voltage was 2.01 kV, ion source temperature was 150 °C, and desolvation gas (nitrogen) temperature was 500 °C. The flow rate of the nebulizer gas (nitrogen) was 1,000 L/h, and the cone hole voltage was 30 V (Gao et al., 2018).

The molecule weight (Mw) and number average molecular weight (Mn) of ONP were concluded by gel permeation chromatography (GPC) with the Breeze system (Waters 1525, USA) accompany with the PL aquagel‐OH MIXED column kept at 30 °C and coupled to the refractive index (RI) detector (Cao et al., 2020)[4]. ONP was made elution with 0.2 M NaNO_3_ and 0.01 M NaH_2_PO_4_ at a flow rate of 1.0 ml/min. The narrow distribution dextran with molecular weights from 100 to 1,214,000 (Mol Wt, Polymer Standards Service, USA) is used to estimate the Mw of ONP (Figure S1). The calibration curve was obtained by plotting the elution volumes against the logarithm of their respective Mw. According to the definition of the International Union of Pure and Applied Chemistry (IUPAC), the polydispersion index (PDI) is calculated as follows: Dm=Mw/Mn (Cao et al., 2020; Wang et al., 2020).

### 2.3 Cell culture

Mouse macrophage cell line RAW264.7 was purchased from Chinese Academy of Sciences Cell Bank (Shanghai, China). RAW264.7 cells were cultured in DMEM containing 10% FBS at 37°C in a 5% CO_2_ incubator. The cell viability was determined by trypan blue dye exclusion method and direct counting with a hemocytometer. RAW264.7 cells (5 × 10^6^ cells) were inoculated into a 25 cm^3^ tissue culture flask and incubated for 24 hr in a 37% humid environment containing 5% CO_2_. The attached cells were incubated with ONP and 1.0 μg/mL LPS in 37% humid environment containing 5% CO_2_ for 24 hr.

### 2.4 Animals

Male mice (C57BL/6), 6 to 8 weeks of age, were purchased from Liaoning Changsheng Biotechnology Co. Ltd (Shengyang, China). Mice were reared under constant temperature and humidity (12 hr light/dark cycles). After acclimating for 2 weeks, the mice were divided into 5 experimental groups (18 in each group): control group (gavage administration of 0.9% saline once a day), ONP group (gavage administration of 800 mg/kg ONP once a day), DDP group (injection of 5.0 mg/kg DDP every other day), DDP‐ONP‐L group (injection of 5.0 mg/kg DDP every other day as well as gavage administration of 200 mg/kg ONP once a day), and DDP‐ONP‐H group (injection of 5.0 mg/kg DDP every other day as well as gavage administration of 800 mg/kg ONP once a day).

On the 14th day, 6 mice in each group were anesthetized with and sacrificed by cervical dislocation to test their biological activity; the remaining mice were used for survival curve analysis, DDP injection was stopped, and ONP was continuously administered. All animal experiments were conducted in accordance with the protocols approved by the Animal Care and Use Committee of Kunming University of Science and Technology (Permit No. SCXK 2015–0001).

### 2.5 Histological analysis

The kidneys of mice washed with PBS were collected. The kidney tissue is treated with 10% paraformaldehyde, ethanol, and xylene, and finally, a wax block containing tissue is made. The waxy block was cut into thin slices (4 μm) and stained with hematoxylin and eosin (H&E). The image was captured by Panorama MIDI (Hungarian 3D HISTECH).

### 2.6 Hematological assays

The blood of the mice was collected and placed in a collection tube containing anticoagulant. After centrifugation at 4,000 rpm for 10 min, serum was collected. The contents of BUN, CRE, Ca, and P in serum were determined with a diagnostic kit.

### 2.7 Biochemical assays of kidney

The kidney tissue was ground into homogenate in ice physiological water. The homogenate was centrifuged at 800 × g for 5 min at 4°C to obtain a crude supernatant. The crude supernatant was further centrifuged at 500 × g for 20 min at 4°C to obtain a pure supernatant, which was used to determine the contents of MDA, SOD, and GSH‐PX.

### 2.8 Real‐time quantitative polymerase chain reaction (qRT‐PCR)

Total RNA of the RAW264.7 cell and kidney tissue was extracted and reverse‐transcribed it into cDNA, and then, qRT‐PCR was carried out on the LightCycler^®^96 Real‐Time PCR system (Roche, Switzerland). The primer sequences are in supplementary materials Table S1 in the support information. Based on the 2^‐ΔΔCt^ method, the β‐actin gene was used as an internal reference.

### 2.9 Western blot analysis

The total protein of renal tissues was extracted, and its concentration was determined. After separating the protein by SDS‐PAGE, it was transferred to a solid‐phase carrier polyvinylidene fluoride (PVDF) membrane. The PVDF membrane was blocked with 5% skimmed milk powder for 2 hr at room temperature. Incubate with primary antibody overnight at 4°C, after that incubate with HRP‐conjugated goat anti‐rabbit or anti‐mouse at room temperature for 1 hr. Using an illumination imaging system (Tanon, China), the gray value of the protein band was detected by ECL kit and Image pro plus 6.0 software.

### 2.10 TUNEL analysis

Kidney tissues were subjected to TUNEL staining using an in situ apoptosis detection kit in order to analyze the apoptosis of renal cells. The waxy block was cut into thin sections (4 μm) and dewaxed according to standard procedures. Place the slides in a plastic jar containing 200 ml 0.1 M citrate buffer, pH 6.0. Apply 750 W (high) microwave irradiation for 1 min. Cool rapidly by immediately adding 80 ml double‐distilled water. Transfer the slides into PBS. Immerse the slides for 30 min in 0.1 M, pH 7.5 Tris‐HCl, containing 3% BSA and 20% normal bovine serum. Rinse the slides twice with PBS. Add 50 μL of TUNEL reaction mixture. Incubate for 60 min at 37°C in a humidified atmosphere in the dark. Rinse slides three times in PBS for 5 min each. Finally, after color development with 3,3’‐diaminobenzidine (DAB), hematoxylin was counter‐stained, and then, the slides were washed 3 times in PBS, dehydrated, transparent, and mounted. Images were captured by a Pannoramic MIDI (3D HISTECH, Hungary). Image pro plus 6.0 software was used to calculate the average value of apoptosis in 10 fields selected randomly for each slice (Mahgoub et al., 2017; Xing et al., 2019).

### 2.11 Statistical analysis

The data are expressed as mean ± *SEM*. One‐way analysis of variance (ANOVA) followed by Tukey–Kramer test was used to analyze statistical comparisons to identify significant differences between groups.

## 3 RESULTS

### 3.1 Analysis of polysaccharide components

**FIGURE 1:**
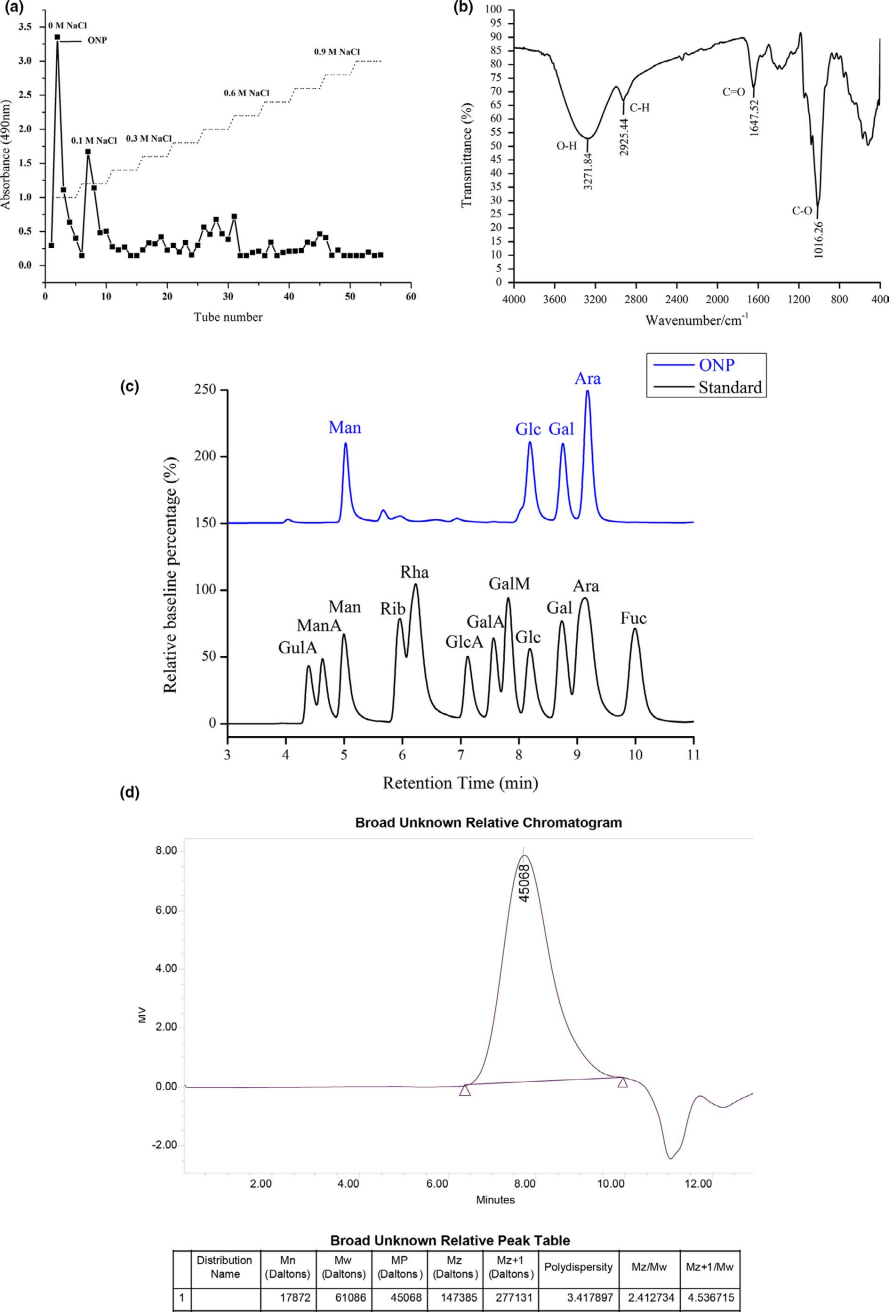
Analysis of polysaccharide components. (a) Elution profiles of crude *O. lanpingensis* polysaccharides using a DEAE‐Sepharose^TM^ Fast Flow (10 cm×60 cm) column for stepwise elution at a flow rate of 1.0 ml/min; (b) the FT‐IR spectrum of ONP; (c) UPLC‐MS analysis of total ion chromatograms (TIC) of ONP monosaccharide composition; (d) gel permeation chromatogram of ONP. ONP, *O. lanpingensis* neutral polysaccharide. Gulonic acid (GulA); mannuronic acid (ManA); mannose (Man); ribose (Rib); rhamnose (Rha); glucuronic acid (GlcA); galacturonic acid (GalA); galactosamine (GalM); glucose (Glc); galactose (Gal); arabinose (Ara); fucose (Fuc)

Crude polysaccharides were dissolved in distilled water and loaded them on the DEAE‐Sepharose^TM^ Fast Flow (10 cm×60 cm) column. Sodium chloride solutions at different concentrations (0 M – 1.0 M NaCl) were applied in gradient elution, and the eluents were examined using the phenol sulfuric acid method (Albalasmeh et al., 2013). As shown in Figure 1a, the *O. lanpingensis* neutral polysaccharide (ONP) was collected.

FT‐IR spectra of carbohydrates provide information on the primary structural characteristics of polysaccharides (Zhao et al., 2019). The FT‐IR spectrum of ONP is shown in Figure 1b. The absorption bands at 3,271.84 cm^−1^, 2,925.44 cm^−1^, 1647.52 cm^−1^, and 1,016.26 cm^‐1^ were characteristic absorption peaks of polysaccharides (Wu et al., 2018). The stretching vibration of the O‐H had continuous strong and broad absorption band at 3,271.84 cm^‐1^. Peak of C‐H was shown at 2,925.44 cm^‐1^. The relatively strong absorption peak at 1,016.26 cm^‐1^ indicated the characteristic vibration of the C‐O bond. The absorption peak at 1647.52 cm^‐1^ was the characteristic vibration peak of the C = O bond. Such results above demonstrated that ONP was one kind of typical carbohydrates.

In addition, after precolumn derivatization of 1‐phenyl‐3‐methyl‐5‐pyrazolone (PMP), the compositions and contents of monosaccharides in ONP were analyzed by UPLC‐MS (Table S2 and Figures S2‐S3). The ion chromatograms of standard monosaccharides and ONP are shown in Figure 1c, which indicated that ONP was a heteropolysaccharide mainly composed of mannose (Man), glucose (Glc), galactose (Gal), and arabinose (Ara) with the molar ratio of 21.9:27.5:19.5:31.1. These four kinds of monosaccharides formed the backbone of ONP.

Mw is the total weight of the sample divided by the weight of the individual molecules, while Mn is the total weight of the sample divided by the number of molecules (Cao et al., 2020). The calculated Mw and Mn of ONP are 6.1 × 10^4^ Da and 1.79 × 10^4^ Da, respectively (Figure 1d). In addition, the PDI of ONP was 3.42, indicating dispersed rather than uniformly sized particles.

### 3.2 ONP relived the inflammatory status of LPS‐induced RAW264.7 macrophage

According to previous studies, DDP‐induced nephrotoxicity could increase the expression levels of pro‐inflammatory factors (iNOS, COX‐2, TNF‐α, and IL‐1β) and decrease the expression level of IL‐10 anti‐inflammatory factors (Gao et al., 2016; Holditch et al., 2019). Therefore, we used the inflammatory experiment of RAW 264.7 cell induced by LPS to confirm whether ONP had anti‐inflammatory activity in vitro (Hwang et al., 2020).

**FIGURE 2:**
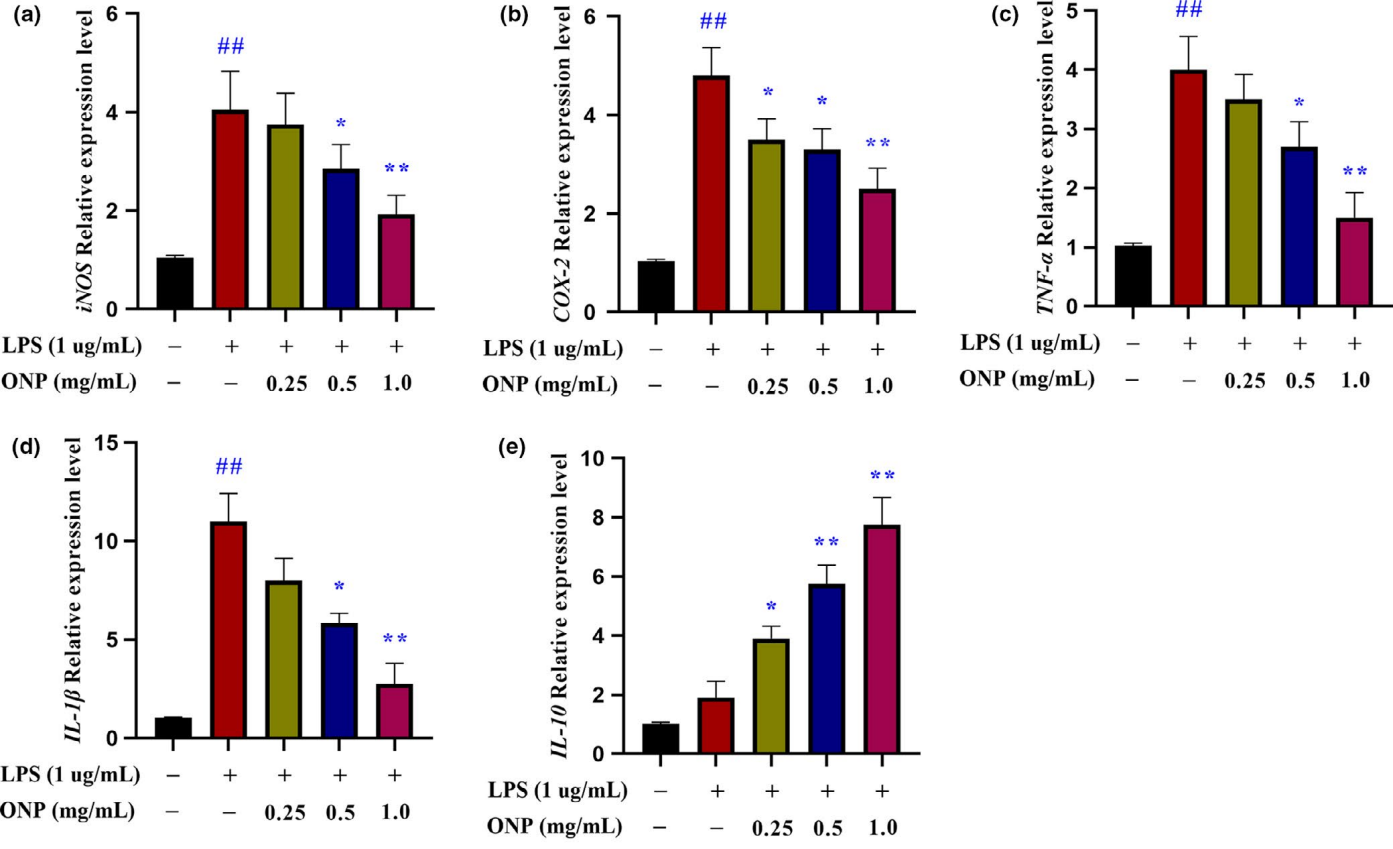
The effect of ONP on 264.7 macrophages induced by LPS. (a‐e) Gene relative expression levels of *iNOS*, *COX‐2*, *TNF‐α*, *IL‐1β*, and *IL‐10*, respectively. Data were presented as the mean ± *SEM* (*n* = 3). LPS, lipopolysaccharide; ONP, *O. lanpingensis* neutral polysaccharide. Compared with the unstimulated cells, ^#^ *p* <.05, ^##^ *p* <.01; compared with the LPS stimulated cells, * *p* <.05, ** *p* <.01, by one‐way ANOVA

Compared with unstimulated cells, the levels of pro‐inflammatory factors, *iNOS*, *COX‐2*, *TNF‐α,* and *IL‐1β*, in RAW264.7 macrophages induced by LPS were significantly higher (Figure 2a‐2d), while ONP treatment attenuated the expression of such pro‐inflammatory factor genes. In addition, ONP treatment resulted in the upregulation of *IL‐10* expression level in RAW264.7 cells (Figure 2e). Therefore, ONP might have anti‐inflammatory activity by alleviating the expression of pro‐inflammatory factors and increasing the expression of anti‐inflammatory factor *IL‐10*.

### 3.3 The survival status of mice treated by DDP or ONP

**FIGURE 3:**
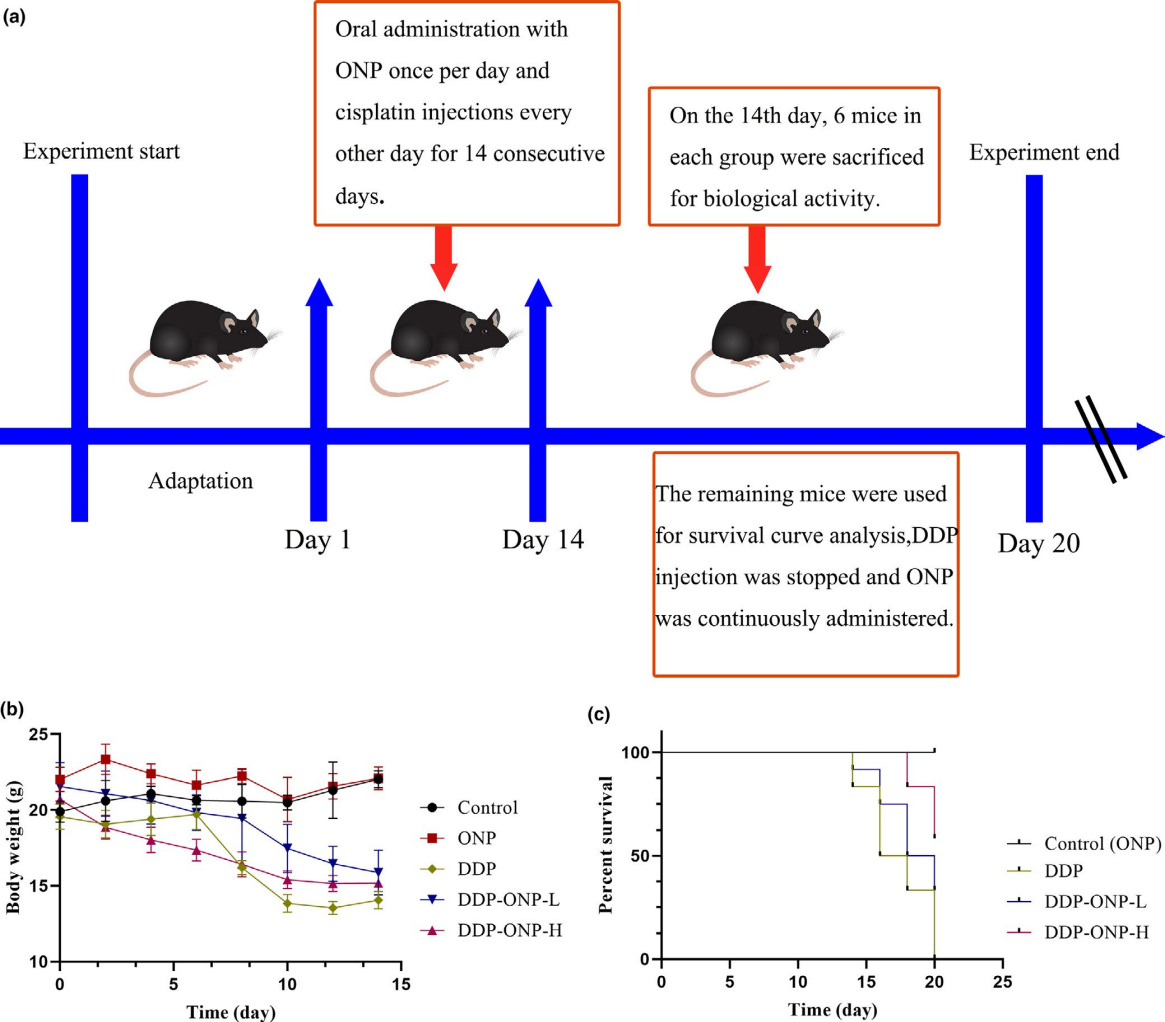
Effect of ONP on the survival status of cisplatin‐treated mice. (a) experimental plan; (b) the body weight changes of mice; (c) survival curves. Data were presented as the mean ± *SEM* (*n* = 12). DDP, cisplatin; ONP, *O. lanpingensis* neutral polysaccharide; ONP‐L, 200 mg/kg ONP; ONP‐H, 800 mg/kg ONP. Compared with the control group, ^#^ *p* <.05, ^##^ *p* <.01; compared with the DDP group, * *p* <.05, ** *p* <.01, by one‐way ANOVA

The experiment was carried out according to Figure 3a. Compared with the control group, DDP treatments resulted in a significant weight loss and a large number of deaths (Figure 3b‐3c). The loss of body weight also happened in DDP‐ONP groups, but the reduced weight was more slowly compared with the DDP group.

In addition, ONP could remarkably increase the survival rate of DDP‐treated mice. All the mice were dead on the 20th day after giving DDP lonely, but the survival rate was increased to 58% in the DDP‐ONP‐H group. Such results indicated that ONP might alleviate the side effects of DDP to some extent. Furthermore, there was no significant difference on the body weight or survival rate between the control group and the ONP group, suggesting that ONP possibly had no obvious toxicity. The clinical application of DDP shows that nephrotoxicity is one of its main side effects and may even lead to death (Casanova et al., 2020; Charlier et al., 2004). Based on the attenuation of body weight loss and the increasing of survival rate in mice, ONP was likely to have the potential to protect the kidneys.

### 3.4 Effects of ONP on morphological and histopathological changes of kidney in DDP‐treated mice

**FIGURE 4:**
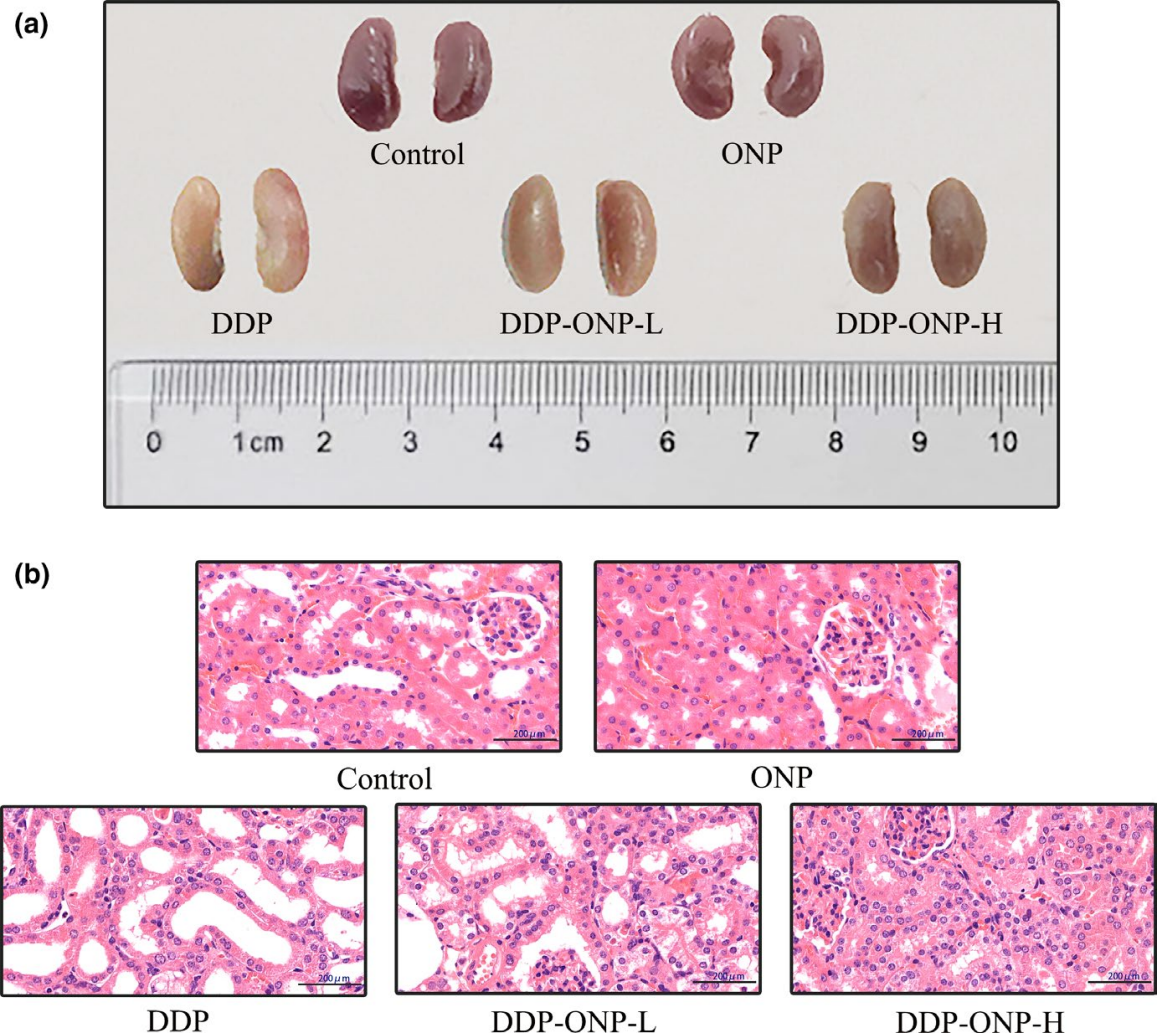
Effects of ONP on morphological and histopathological changes of kidney in DDP‐treated mice. (a) Morphological changes of the kidneys; (b) histological changes of kidney tissues stained with H&E. DDP, cisplatin; ONP, *O. lanpingensis* neutral polysaccharide; ONP‐L, 200 mg/kg ONP; ONP‐H, 800 mg/kg ONP. Scale bar, 200 μm; magnification, 400×

The morphological changes of the kidneys are shown in Figure 4a, which indicated that the appearance color was changed from dark red to white in the DDP treatment compared with the control group; nevertheless, ONP treatments could slow such a harmful change. Next, we performed pathological analysis of renal tissues.

The pathological detection of the DDP group clearly showed massive necrosis of the renal tubules and glomeruli (Figure 4b), but such pathological damage was decreased with the treatments of ONP in DDP‐ONP‐L and DDP‐ONP‐H groups. Furthermore, there was no significant difference between ONP group and control group. Overall, ONP treatment attenuated the DDP‐induced kidney damage.

### 3.5 ONP inhibited the renal function impaired by DDP

**FIGURE 5:**
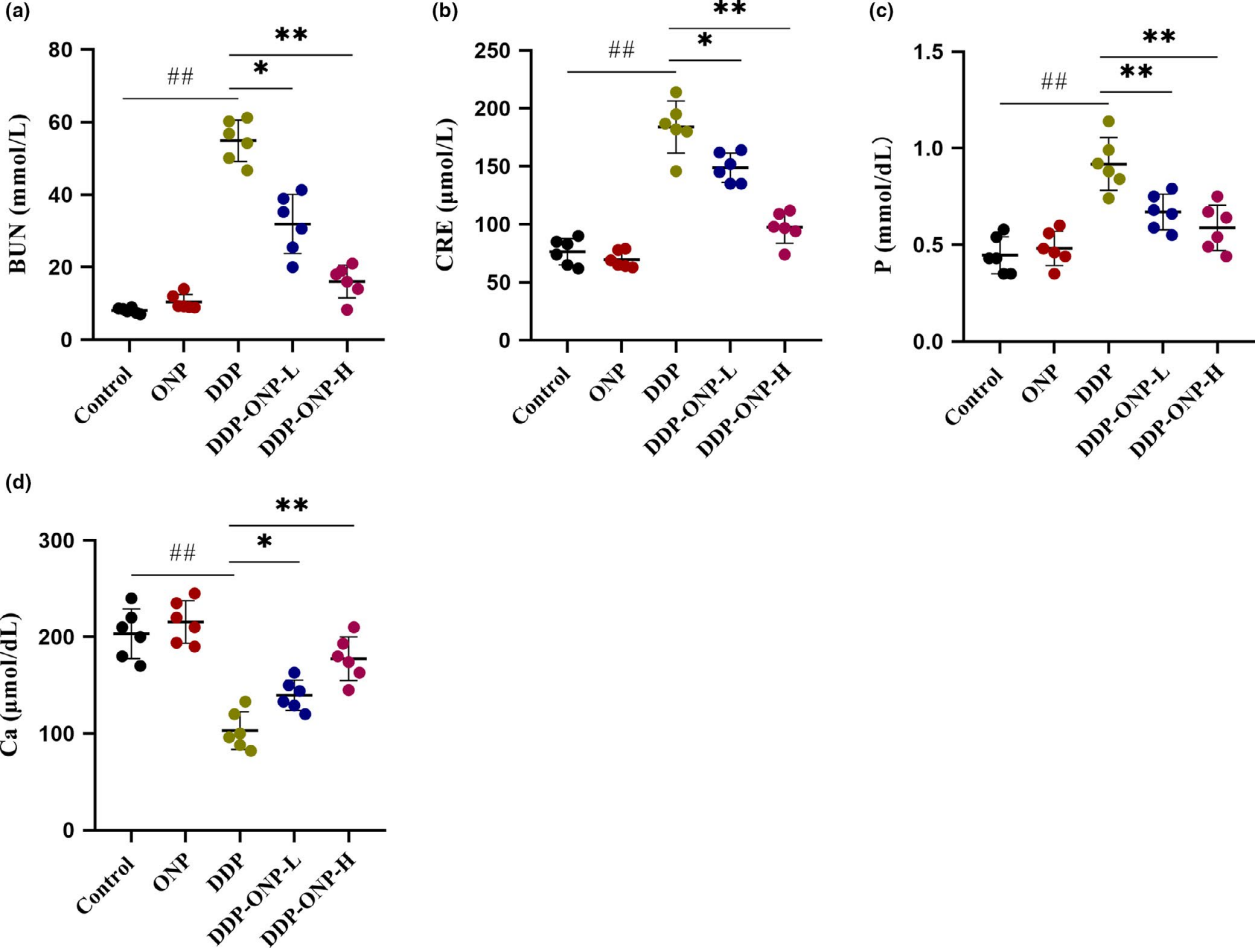
ONP recovered the renal function impaired by cisplatin. (a) The level of blood urea nitrogen (BUN); (b) the level of blood creatinine (CRE); (c) the level of blood phosphorus (P); (d) the level of blood calcium (Ca). Data were presented as the mean ± *SEM* (*n* = 6). DDP, cisplatin; ONP, *O. lanpingensis* neutral polysaccharide; ONP‐L, 200 mg/kg ONP; ONP‐H, 800 mg/kg ONP. Compared with the control group, ^#^ *p* <.05, ^##^ *p* <.01; compared with the DDP group, * *p* <.05, ** *p* <.01, by one‐way ANOVA

The concentrations of BUN, CRE, P, and Ca in serum are important biochemical indicators for evaluating renal function (Ma et al., 2017; Meurer and Hocherl, 2019). In this study, compared with the control group, the contents of BUN, CRE, and P in the DDP group were elevated more than 4, 1, and 1 times, respectively; meanwhile, the content of Ca in the DDP group was declined to about 50% (Figure 5). Moreover, ONP treatment diminished these pathological changes with a dose‐dependent relationship. All the serum biochemical indexes referred to above were almost remained normal in the DDP‐ONP‐H group.

### 3.6 ONP reduced oxidative stress and inhibited the expression of inflammatory factors in the kidney

**FIGURE 6:**
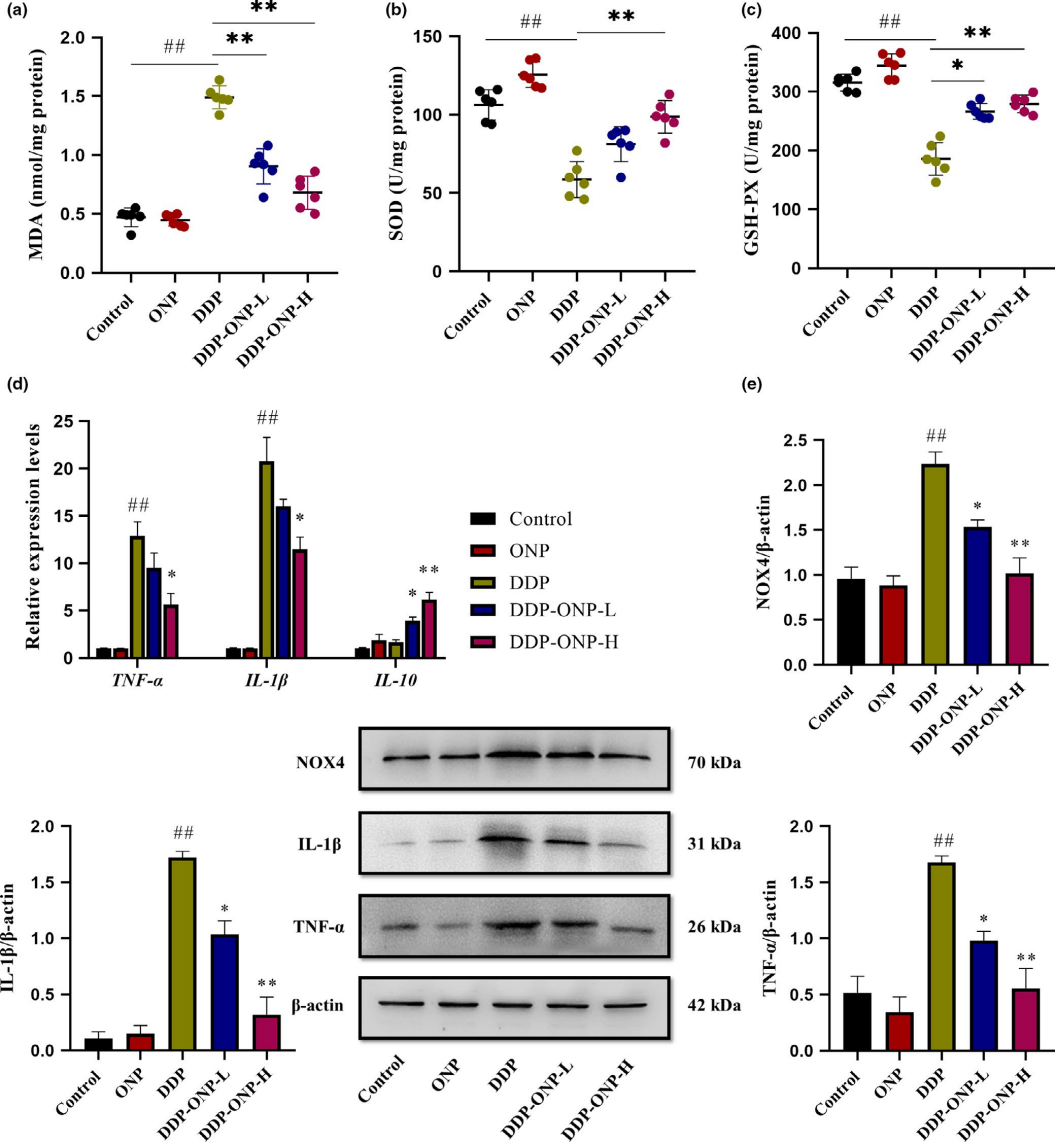
ONP reduced oxidative stress and inhibited the expression of inflammatory factors in the kidney. (a) Malondialdehyde (MDA); (b) superoxide dismutase (SOD); (c) glutathione peroxidase (GSH‐PX); (d) gene relative expression levels of *TNF‐α*, *IL‐1β*, and *IL‐10*; (E) protein expressions of NOX4, IL‐1β, TNF‐α. Data were presented as the mean ± *SEM* (*n* = 6). DDP, cisplatin; ONP, *O. lanpingensis* neutral polysaccharide; ONP‐L, 200 mg/kg ONP; ONP‐H, 800 mg/kg ONP. Compared with the control group, ^#^ *p* <.05, ^##^ *p* <.01; compared with the DDP group, * *p* <.05, ** *p* <.01, by one‐way ANOVA

MDA is considered to be a sign of oxidative stress, and endogenous antioxidant enzymes SOD and GSH‐PX are the key factors to maintain redox balance (Kim, Song, et al., 2020; Tsikas, 2017). As shown in Figure 6a‐6c, compared with the control group, the MDA and antioxidant enzyme (SOD and GSH‐PX) levels increased and decreased significantly in the DDP group, respectively, which indicated that oxidative stress was induced by DDP in the kidneys. In contrast, ONP could inhibit MDA production and enhance antioxidant enzyme levels in the DDP‐ONP groups thus relieving the oxidative stress.

NOX4 is a key enzyme that can catalyze the generation of ROS from oxygen in several kinds of renal cells including endothelial cells, vascular smooth muscle cells, and epithelial cells of renal tubules; therefore, NOX4 plays important roles in renal oxidative stress and kidney injury (Gao et al., 2016; Qin et al., 2018). Some studies have proved that the expression levels of pro‐inflammatory factors such as TNF‐α and IL‐1β could be enhanced with the increased expression of NOX4 (Gao et al., 2016; Ma et al., 2017)\.

As shown in Figure 6d‐6e, the expression levels of NOX4, TNF‐α, and IL‐1β in the DDP group were elevated compared with the control group, which indicated that inflammatory response might be activated by DDP in the renal tissues. On the contrary, ONP could inhibit NOX4 production and the expression levels of TNF‐α and IL‐1β. In addition, ONP also increased the expression of the anti‐inflammatory factor *IL‐10* gene.

### 3.7 ONP relieved the apoptosis of renal cells caused by DDP

**FIGURE 7:**
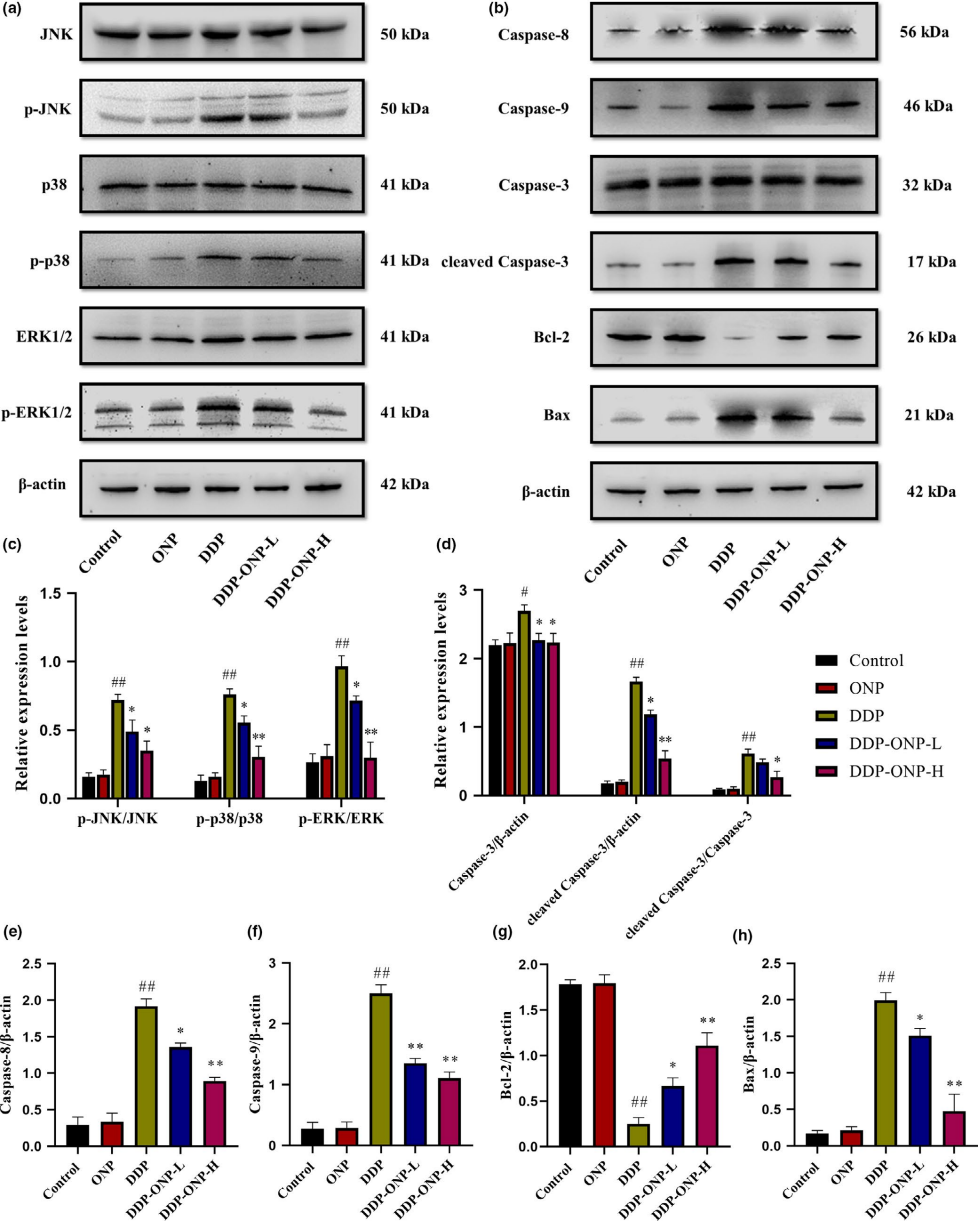
The expression levels of pro‐apoptosis proteins. (a‐b) Protein representative bands; (c‐h) quantitative analysis of scanning densitometry for MAPK, caspase‐3, cleaved caspase‐3, caspase‐8, caspase‐9, Bcl‐2, and Bax. Data were presented as the mean ± *SEM* (*n* = 6). DDP, cisplatin; ONP, *O. lanpingensis* neutral polysaccharide; ONP‐L, 200 mg/kg ONP; ONP‐H, 800 mg/kg ONP. Compared with the control group, ^#^ *p* <.05, ^##^ *p* <.01; compared with the DDP group, * *p* <.05, ** *p* <.01, by one‐way ANOVA

In order to evaluate the degree of apoptosis in kidneys, the protein expression levels of the JNK, p‐JNK, p38, p‐p38, ERK1/2, p‐ERK1/2, caspase‐8, caspase‐9, caspase‐3, cleaved caspase‐3, the anti‐apoptotic factor Bcl‐2, and the pro‐apoptotic factor Bax were determined (Figure 7). The results showed that DDP treatment significantly increased the expression levels of p‐JNK, p‐p38, p‐ERK1/2, caspase‐8, caspase‐9, caspase‐3, cleaved caspase‐3, and Bax, meanwhile decreased the expression level of Bcl‐2 compared with the control group. Notably, these changes could be effectively reversed by ONP treatment.

**FIGURE 8:**
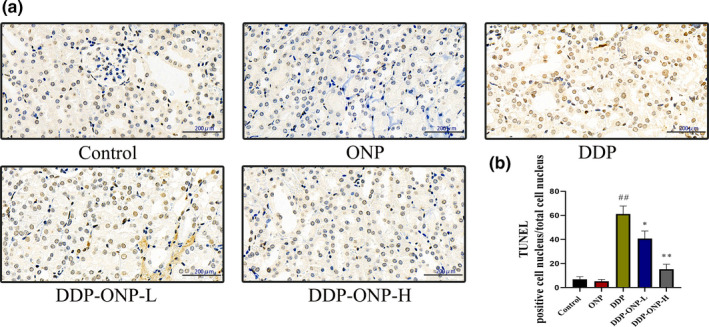
ONP treatments inhibited the apoptosis of renal cells. (a) TUNEL of kidney tissues; (b) statistics of TUNEL stain assay. Apoptosis cells were stained in brown. Data were presented as the mean ± *SEM* (*n* = 6). DDP, cisplatin; ONP, *O. lanpingensis* neutral polysaccharide; ONP‐L, 200 mg/kg ONP; ONP‐H, 800 mg/kg ONP. Compared with the control group, ^#^ *p* <.05, ^##^ *p* <.01; compared with the DDP group, * *p* <.05, ** *p* <.01, by one‐way ANOVA. Scale bar, 200 μm; magnification, 400

Furthermore, the apoptosis of the kidney tissues was quantified by the TUNEL assay staining which directly revealed renal cell apoptosis after DDP exposure. It was interesting that ONP treatment obviously reduced the number of TUNEL‐positive cells. Such results indicated that DDP significantly induced apoptosis and ONP inhibited the apoptosis caused by DDP (Figure 8).

## 4 DISCUSSION

In recent years, natural product polysaccharides, especially fungal polysaccharides, have shown unique pharmacological effects in immune regulation, anti‐inflammatory, anti‐oxidation, anti‐aging, and disease prevention such as polysaccharides from *Ganoderma lucidum*, *Flammulina velutipes,* or *Cordyceps militaris* could prevent and treat diabetic nephropathy (Geng et al., 2020; Kim, Wu, et al., 2020; Li et al., 2019; Lin et al., 2016).

This study extracted a kind of neutral polysaccharide from *O. lanpingensis*, which is composed of four monosaccharides: mannose, glucose, galactose, and arabinose. Polysaccharides have been reported to possess many bioactivities. *Pleurotus djamor* mycelial polysaccharide is mainly composed of mannose and glucose, which has antioxidant and anti‐aging effects; *Flammulina velutipes* polysaccharide can inhibit the development of diabetes, which is composed of ribose, arabinose, xylose, galactose, and glucose (Li et al., 2019; Lin et al., 2016). So far, the potential pharmacological activities are hard to be estimated based on monosaccharides composition of polysaccharides, even though the fungal polysaccharides have so many functional activities.

The inflammatory status was induced by LPS in RAW 264.7 cells. It is known that LPS stimulates macrophages to express more nitric oxide synthase (iNOS) and cyclooxygenase‐2 (COX‐2) to produce NO and prostaglandin E2 (Hwang et al., 2020)[20]. Moreover, iNOS and COX‐2 activate the expression levels of several pro‐inflammatory mediators, such as TNF‐α and IL‐1β. In our study, *O. lanpingensis* neutral polysaccharide (ONP) inhibited the upregulated expression levels of *iNOS*, *COX‐2*, *TNF‐α,* and *IL‐1β* induced by LPS, which also increased the expression level of *IL‐10* in a dose‐dependent manner in RAW 264.7 cells. IL‐10 is a multifunctional cytokine that regulates cell growth and differentiation, participates in inflammatory reactions, and is recognized as an inflammation inhibitor (Ip et al., 2017). Based on these results and the ethnic traditional usage of *O. lanpingensis* in the treatment of urinary system diseases, it is valuable to explore whether the ONP could alleviate DPP‐induced nephrotoxicity.

In general, the clinical side effects of DDP mainly include nephrotoxicity, gastrointestinal toxicity, neurotoxicity, and ototoxicity, of which nephrotoxicity is particularly significant and may even lead to death (Arany and Safirstein, 2003; Oun and Rowan, 2017). DDP is mainly metabolized by glomerular filtration and proximal renal tubular epithelial cells, thus to accumulate DDP in the kidney to a large amount. The concentration of DDP in the renal tissues is about five times of that in the blood and such side effect eventually leads to renal dysfunction (Volarevic et al., 2019). A recent study revealed that AKI was a common multifactorial complication in the later stages of the course of severe COVID‐19 patients, and the pathological characteristics of such AKT were similar to the AKT induced by DDP (Xia, Wen, et al., 2020). The predominant pathologic finding of COVID‐19 patients was acute tubular injury, which eventually led to kidney organ failure and even death. In our study, ONP could reduce the rate of weight loss and increase the survival rate when DDP‐induced AKT was occurred in mice. Maybe there is a potential application on severe COVID‐19 patients by ONP, but it is just a hypothesis.

Morphological and pathological sections of mouse kidney preliminarily proved that ONP could alleviate nephrotoxicity. DDP exposure can induce the damage of renal vasculature and result in the decline of glomerular filtration rate (Arany et al., 2003)[2]. It is known that the kidney is the main excretory organ of urea. After urea is filtered by the glomerulus, it can be reabsorbed by the renal tubules. When renal function is impaired, the flow rate of urine decreases, urea is reabsorbed more by the renal tubules, and the BUN content in the blood increases (Ma et al., 2017). CRE is a product of muscle metabolism. In muscles, creatine slowly forms CRE through an irreversible nonenzymatic dehydration reaction, and then, CRE is released into the blood and excreted in the urine. When the glomerulus is damaged, the ability of filtering CRE by glomerulus will be decreased, and the content of CRE in the serum will be increased (Smith, 1988). Therefore, the concentrations of BUN and CRE are important biochemical markers for assessing glomerular filtration rate and renal function. In addition, renal failure results in the enhancement and reduction of the expression levels of P and Ca transporters, respectively (Meurer and Hocherl, 2019). In this study, ONP alleviated the increasing levels of BUN, CRE, and P, as well as inhibiting the decreasing level of Ca in the serum after the mice were treated by DDP, so that ONP could protect renal function to some extent.

The hydrated form of DDP can easily lead to the increased level of reactive oxygen species (ROS) in cells (Karasawa and Steyger, 2015). ROS is a class of molecules with highly reactive, which can attack vicinal biomolecules including proteins, DNA, and lipids. The lipids are oxidized to the finally form MDA (Tsikas, 2017). Therefore, MDA is regarded as a marker of oxidative stress (Olatunji et al., 2016). On the other hand, the endogenous antioxidant enzymes, SOD and GSH‐PX, are key factors to clear ROS in the organism during oxidative stress occurrence in order to maintain the redox equilibrium (Kim, Song, et al., 2020). Previous studies have shown that DDP‐induced inflammation might be NOX4‐dependent, and TNF‐α and IL‐1β levels would be increased (Gao et al., 2016). TNF‐α stimulates the production of genotoxic molecules (NO and ROS) and causes DNA damage and mutations (Hussain et al., 2003). IL‐1β is a trigger of inflammation inducing mediators, thus leading to kidney damage (Dinarello et al., 2012). Our results showed that ONP could attenuate nephrotoxicity through inhibiting the oxidative stress, which also resonated with the similar mechanisms reported previously (Shen et al., 2020). The reduced oxidative stress and secretion of pro‐inflammatory factors probably served as certain mechanisms of reducing cisplatin nephrotoxicity by ONP.

Under the oxidative stress and inflammation conditions, excessive ROS and pro‐inflammatory cytokines could damage cellular proteins, lipids, and DNA, leading to apoptosis of cells (Dasari and Tchounwou, 2014; Florea and Busselberg, 2011). Generally, the pathways of apoptosis are mainly divided into extrinsic pathway and intrinsic pathway (Nunez et al., 1998). TNF‐α mediates caspase‐8 to participate in the extrinsic pathway (Dasari and Tchounwou, 2014; Herbein and O'Brien, 2000). Cell stress induced by DNA damage drives mitochondria to release cytochrome c to activate caspase‐9, which is an intrinsic apoptosis pathway. Moreover, Bcl‐2 family proteins participate in the intrinsic apoptosis pathway by regulating the release of mitochondrial cytochrome c (Ghosh, 2019). Both caspase‐8 and caspase‐9 can activate caspase‐3 by proteolytic cleavage to perform cellular apoptosis (Thornberry and Lazebnik, 1998). In addition, mitogen‐activated protein kinases (MAPK) are a family of structurally related serine/threonine protein kinases that coordinate various extracellular signals to regulate cell growth and survival (Chang and Karin, 2001). Previous studies have shown that TNF‐α and IL‐1β are the main conditions involved in activating JNK, p38, and ERK1/2; moreover, MAPK activation can promote cell apoptosis (Herbein and O'Brien, 2000; Johnson and Lapadat, 2002; Ning et al., 2020; Qi et al., 2019).

DDP cytotoxic mode of action is mediated by its interaction with DNA to form DNA adducts, primarily intrastrand crosslink adducts, which activate several signal transduction pathways, including Bcl‐2 family proteins, caspase family proteins, and MAPK, and culminate in the activation of apoptosis (Siddik, 2003). Notably, our results showed that ONP effectively inhibits the activations of Bcl‐2 family proteins, caspase family proteins, and MAPK in the kidneys of mice, so that to alleviate DDP‐induced nephrotoxicity (Figure 7). Furthermore, the result of TUNEL was consistent with the changes of apoptosis proteins in this study, thus indicating that ONP exactly weakened the apoptosis of renal cells induced by DDP.

## 5 CONCLUSION

**FIGURE 9:**
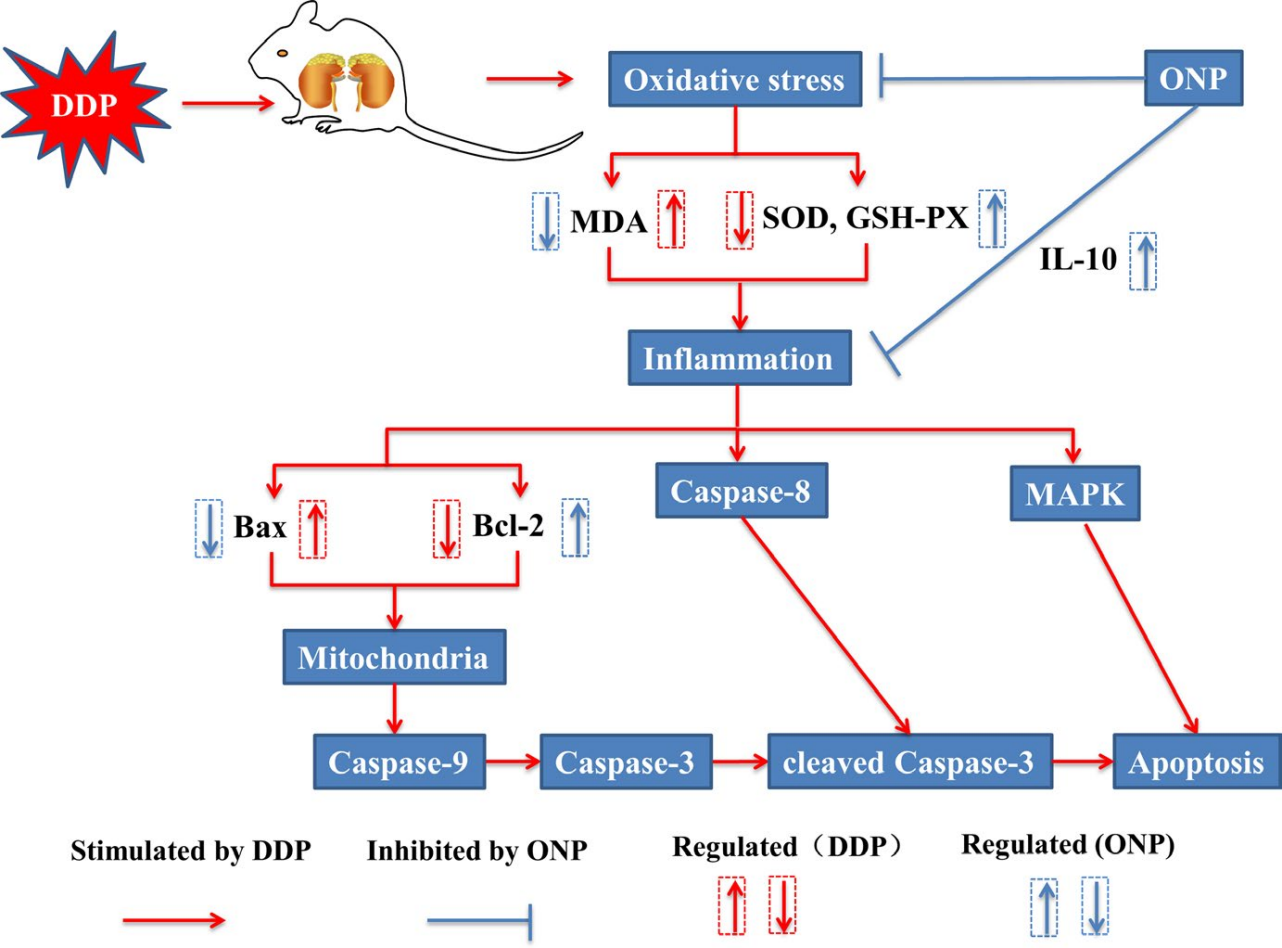
Schematic model of ONP attenuating DDP‐induced nephrotoxicity. DDP, cisplatin; ONP, *O. lanpingensis* neutral polysaccharides. Red lines represented the possible pathway of DDP‐induced renal toxicity in mice. Blue lines represented the possible pathway for ONP to alleviate nephrotoxic in mice

In conclusion, this study revealed the protective effects of ONP on DDP‐induced renal toxicity. Our findings indicated that ONP attenuated DDP nephrotoxicity mainly by reducing oxidative stress, inflammation, and apoptosis in the kidneys, underscoring the potential of ONP supplementation to improve DDP chemotherapy (Figure 9).

## CONFLICT OF INTEREST

The authors declare no conflict of interest and all data can be used.

## Supporting information

Supporting Information

## Data Availability

All data, models, and code generated or used during the study appear in the submitted article.
