# Supplementary material for: A neutral polysaccharide from Ophiocordyceps lanpingensis restrains cisplatin‐induced nephrotoxicity
Source: Food Sci Nutr. 2021 May 11;9(7):3602–16. doi: 10.1002/fsn3.2317 (PMC8269674; doi:10.1002/fsn3.2317)
Supplement: Supplementary file 1 — Supporting Information [file FSN3-9-3602-s001.docx]

**Supplementary Data**

**Table S1.** Primer sequences used in qRT-PCR.

| Gene | GenBank number | Forward | Reverse |
| --- | --- | --- | --- |
| *iNOS* | NC_000077.7 | GAATAATGCCAGATCAGAAACCTACCCA | ACCAAGCTCATGCGGCCTCCT |
| *COX-2* | NC_010339.1 | GGCCTACCCATTCCAACTTGGTCTAC | GTTTCAACTTCTTGTGCGTCTATTGTGCT |
| *β-actin* | NC_000071.6 | GCTGTATTCCCCTCCATCGTG | CACGGTTGGCCTTAGGGTTCAG |
| *TNF-α* | NC_000083.6 | TGTCTACTCCCAGGTTCTCTTCAAGG | TGACGGCAGAGAGGAGGTTGAC |
| *IL-1β* | NC_000068.7 | ACTGTCTGTATAGCCGCTGACATCTA | GCATTAGGAAGATGCTCTGGAAGGAA |
| *IL-10* | NC_000067.7 | GAAGCAGCACCAGCATAGAGAGC | CACCTGTGTCAACCCTTCCTTTC |

**Table S2.** Mass spectrometry analysis parameters of monosaccharides.

| Sample | Number | Name | Trace (m/z) | RT (min) | Molar ratio |
| --- | --- | --- | --- | --- | --- |
| Standard | 1 | Gulonic acid (GulA) | 525 | 4.39 | -- |
| Standard | 2 | Mannuronic acid (ManA) | 525 | 4.627 | -- |
| Standard | 3 | Glucuronic acid (GlcA) | 525 | 7.119 | -- |
| Standard | 4 | Galacturonic acid (GalA) | 525 | 7.564 | -- |
| Standard | 5 | Mannose (Man) | 511 | 4.995 | -- |
| Standard | 6 | Glucose (Glc) | 511 | 8.186 | -- |
| Standard | 7 | Galactose (Gal) | 511 | 8.736 | -- |
| Standard | 8 | Galactosamine (GalM) | 510 | 7.818 | -- |
| Standard | 9 | Rhamnose (Rha) | 495 | 6.167 | -- |
| Standard | 10 | Fucose (Fuc) | 495 | 9.99 | -- |
| Standard | 11 | Ribose (Rib) | 481 | 5.953 | -- |
| Standard | 12 | Arabinose (Ara) | 481 | 9.171 | -- |
| OLP | 1 | Mannose (Man) | 511 | 5.023 | 21.9 |
| OLP | 2 | Glucose (Glc) | 511 | 8.192 | 27.5 |
| OLP | 3 | Galactose (Gal) | 511 | 8.753 | 19.5 |
| OLP | 4 | Arabinose (Ara) | 481 | 9.176 | 31.1 |


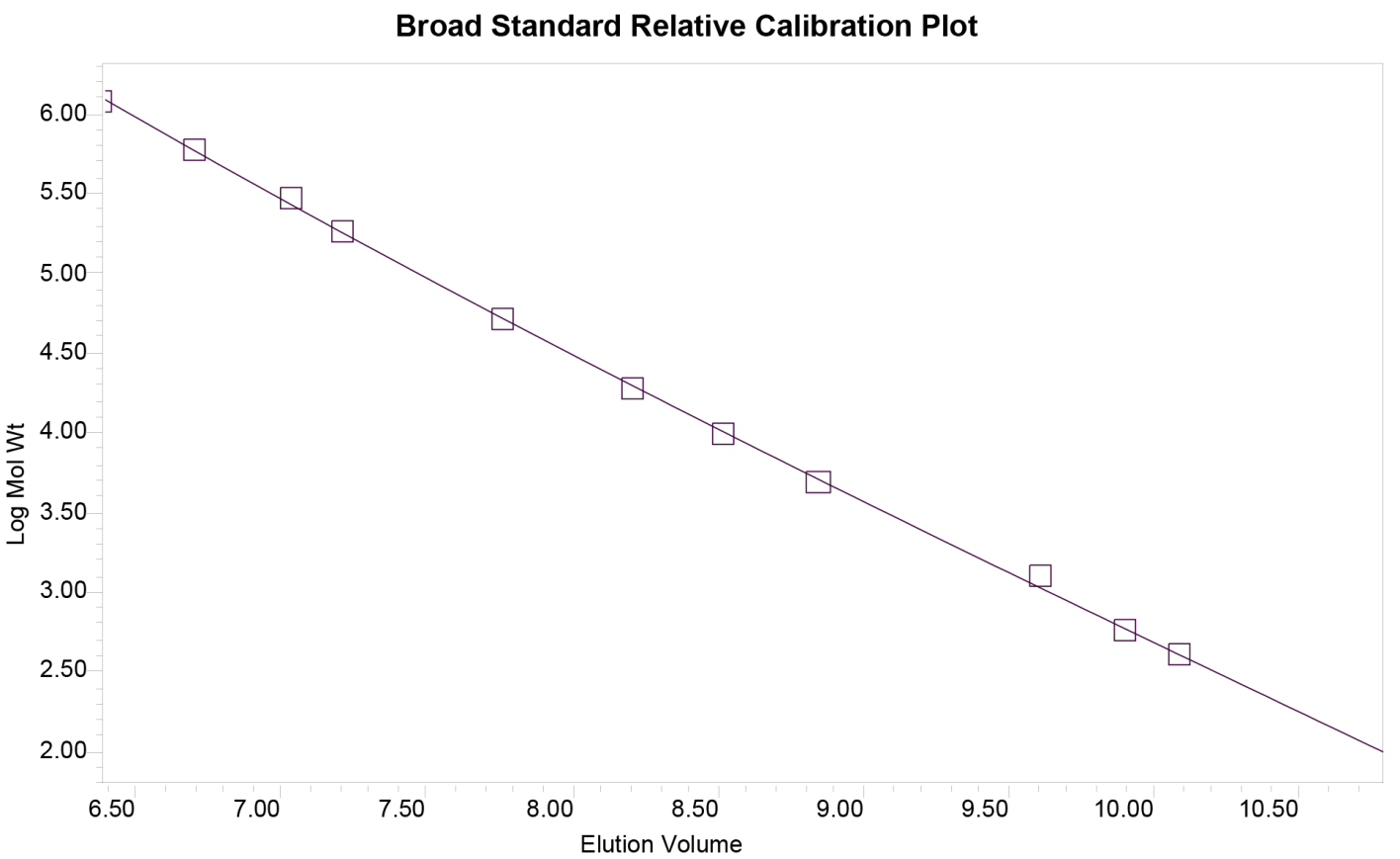


**Figure S1** Standard curve of dextran 100 to 1,214,000 molecular weight (Mw).


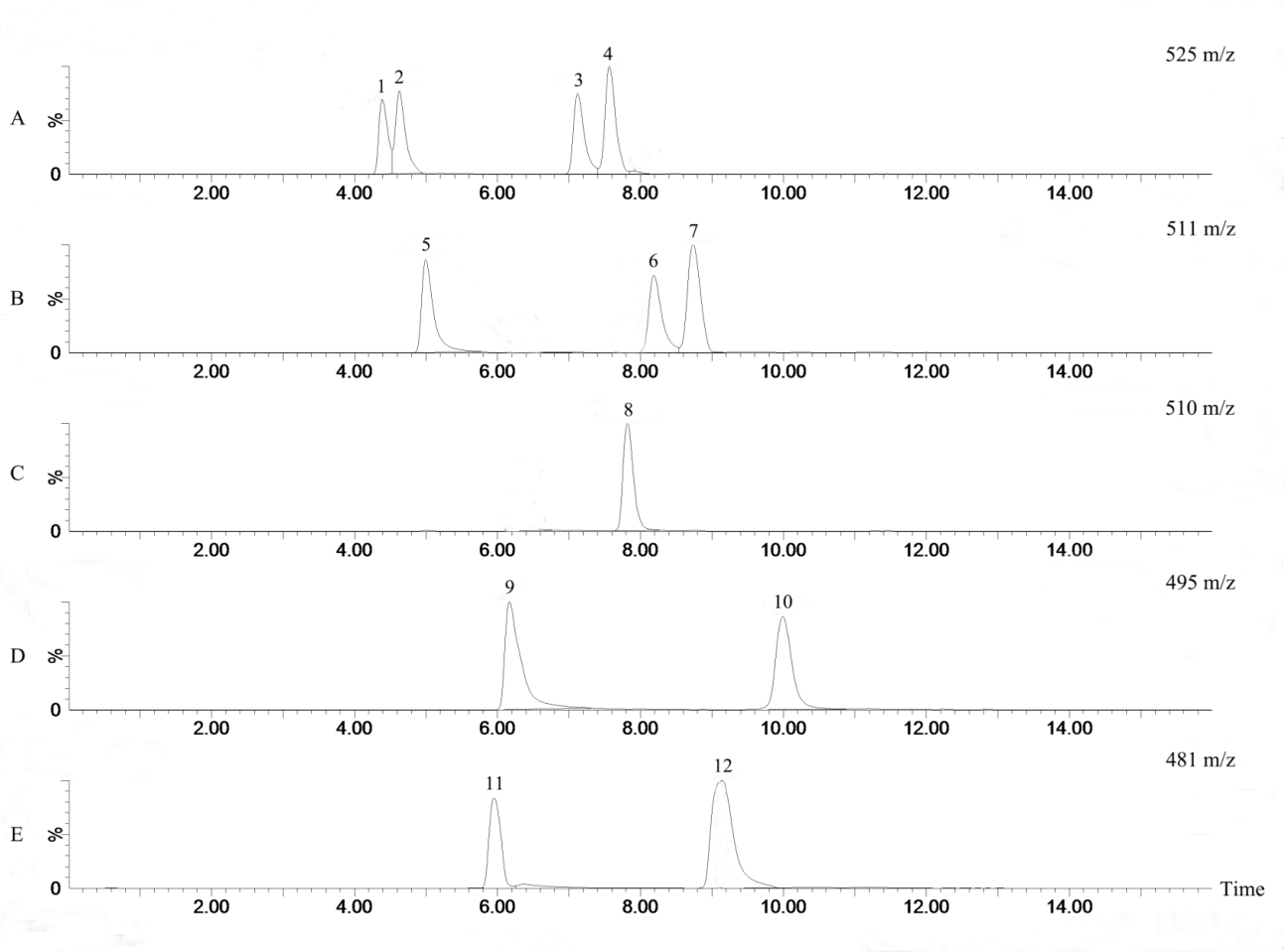


**Figure S2** UPLC-MS chromatograms of standards. (A) 1, Gulonic acid (GulA); 2, Mannuronic acid (ManA); 3, Glucuronic acid (GlcA); 4, Galacturonic acid (GalA). (B) 5, Mannose (Man); 6, Glucose (Glc); 7, Galactose (Gal). (C) 8, Galactosamine (GalM). (D) 9, Rhamnose (Rha); 10, Fucose (Fuc). (E) 11, Ribose (Rib); 12, Arabinose (Ara).

**
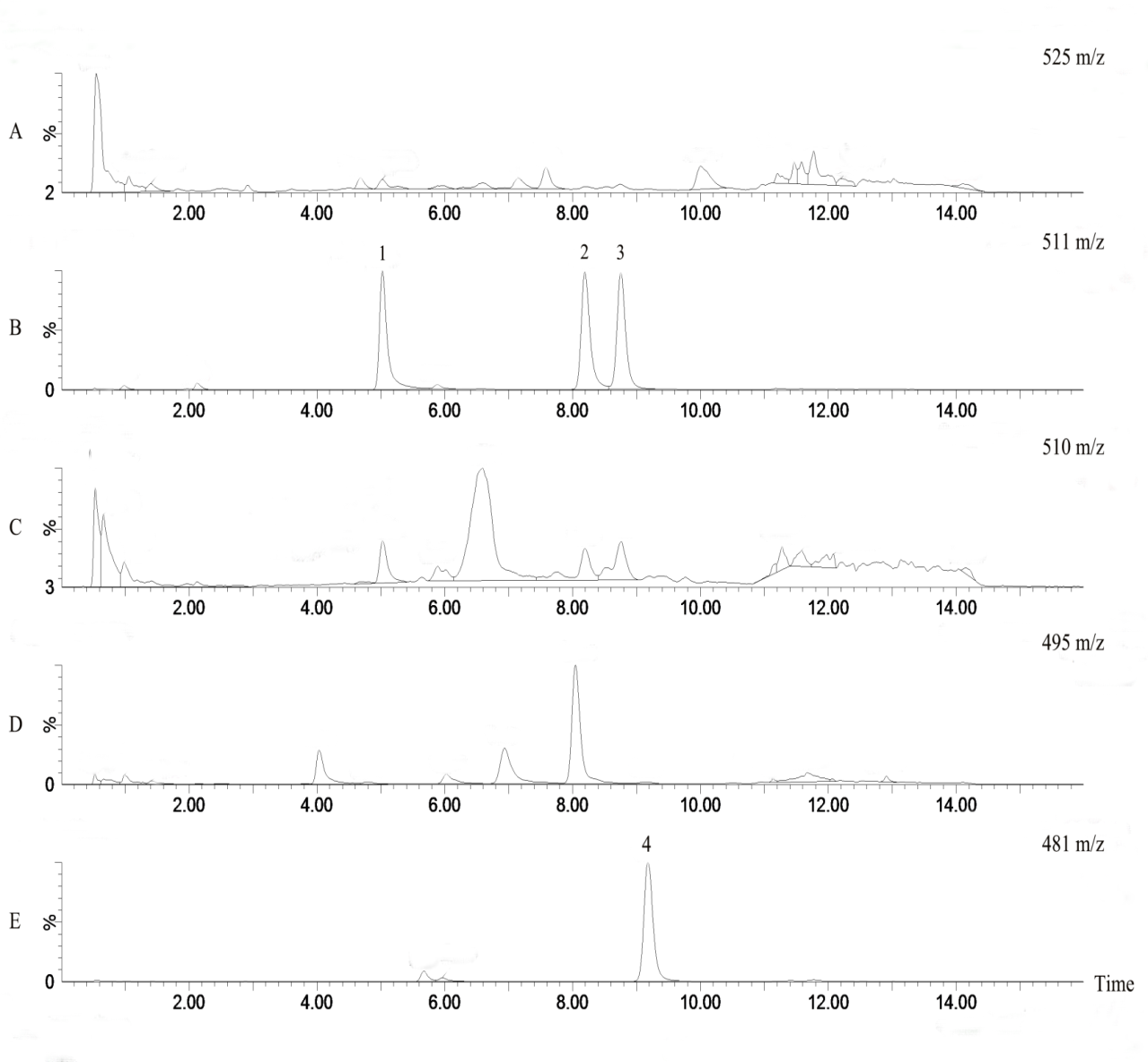
**

**Figure S3** UPLC-MS chromatograms of OLP. (B) 1, Mannose; 2, Glucose; 3, Galactose. (E) 4, Arabinose.
